# Tetra­ethyl­ammonium tricarbonyl­chlorido(isoquinoline-1-carboxyl­ato-κ^2^ *N*,*O*)technetate(I)

**DOI:** 10.1107/S160053680802713X

**Published:** 2008-08-30

**Authors:** Helmut W. Schmalle, Roger Alberto

**Affiliations:** a Anorganisch-Chemisches Institut der Universität Zürich, Winterthurerstrasse 190, CH-8057 Zürich, Switzerland

## Abstract

The asymmetric unit of the title compound, (C_8_H_20_N)[Tc(C_10_H_6_NO_2_)Cl(CO)_3_], consists of two crystallographically independent technetium complexes related *via* a pseudo-inversion centre and two tetra­ethyl­ammonium cations. The Tc atoms have slightly distorted octa­hedral coordination geometries, and they are linked with the cations by inter­molecular C—H⋯O and C—H⋯Cl hydrogen-bonding contacts, forming two-dimensional columns, which lie approximately parallel to (001) in the crystal structure. The isochinolate (isoquinoline-1-carboxyl­ate) ligands link the columns by partial π–π stacking [centroid–centroid distance 4.3733 (11) Å], forming a three-dimensional network structure.

## Related literature

For related literature, see: Alberto *et al.* (1995 ▶, 1996 ▶); Waibel *et al.* (1999 ▶); Rattat *et al.* (2001 ▶); Marsh (1995 ▶); Marsh *et al.* (2002 ▶); Desiraju *et al.* (1991 ▶); Etter *et al.* (1990 ▶); Desiraju & Steiner, (1999 ▶); Bernstein *et al.* (1995 ▶); Steiner & Saenger, 1993 ▶).
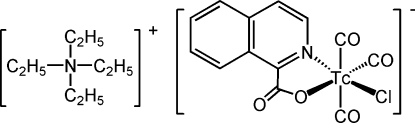


## Experimental

### 

#### Crystal data

(C_8_H_20_N)[Tc(C_10_H_6_NO_2_)Cl(CO)_3_]
*M* *_r_* = 520.80
Triclinic, 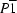

*a* = 11.7657 (14) Å
*b* = 12.7481 (14) Å
*c* = 17.1855 (18) Å
α = 102.878 (12)°
β = 109.624 (12)°
γ = 99.052 (13)°
*V* = 2290.0 (5) Å^3^
*Z* = 4
Mo *K*α radiation
μ = 0.78 mm^−1^
*T* = 193 (2) K
0.77 × 0.48 × 0.19 mm

#### Data collection

Stoe IPDS diffractometer
Absorption correction: numerical (Coppens *et al.*, 1965 ▶) *T*_min_ = 0.670, *T*_max_ = 0.828
29670 measured reflections
12462 independent reflections
9515 reflections with *I* > 2σ(*I*)
*R*_int_ = 0.058

#### Refinement

*R*[*F* ^2^ > 2σ(*F* ^2^)] = 0.028
*wR*(*F* ^2^) = 0.067
*S* = 1.00
12462 reflections
541 parameters
H-atom parameters constrained
Δρ_max_ = 0.54 e Å^−3^
Δρ_min_ = −1.17 e Å^−3^

### 

Data collection: *IPDS Software* (Stoe & Cie, 1997 ▶); cell refinement: *IPDS Software*; data reduction: *X-RED* (Stoe & Cie, 1997 ▶); program(s) used to solve structure: *SHELXS97* (Sheldrick, 2008 ▶); program(s) used to refine structure: *SHELXL97* (Sheldrick, 2008 ▶); molecular graphics: *PLATON* and *PLUTON* (Spek, 2003 ▶); software used to prepare material for publication: *SHELXL97*.

## Supplementary Material

Crystal structure: contains datablocks global, I. DOI: 10.1107/S160053680802713X/hb2781sup1.cif

Structure factors: contains datablocks I. DOI: 10.1107/S160053680802713X/hb2781Isup2.hkl

Additional supplementary materials:  crystallographic information; 3D view; checkCIF report

## Figures and Tables

**Table 1:** Selected bond lengths (Å)

Tc1—C21	1.9045 (18)
Tc1—C31	1.913 (2)
Tc1—C11	1.916 (2)
Tc1—O41	2.1293 (12)
Tc1—N51	2.1778 (15)
Tc1—Cl1	2.4822 (6)
Tc2—C22	1.9060 (19)
Tc2—C32	1.907 (2)
Tc2—C12	1.913 (2)
Tc2—O42	2.1317 (12)
Tc2—N52	2.1714 (15)
Tc2—Cl2	2.4980 (6)

**Table 2:** Hydrogen-bond geometry (Å, °)

*D*—H⋯*A*	*D*—H	H⋯*A*	*D*⋯*A*	*D*—H⋯*A*
C151—H15*A*⋯O51	0.99	2.38	3.328 (2)	161
C171—H17*A*⋯O31^i^	0.99	2.50	3.482 (3)	172
C191—H19*B*⋯O41	0.99	2.40	3.360 (2)	163
C192—H19*C*⋯O42^ii^	0.99	2.56	3.507 (2)	160
C192—H19*C*⋯O52^ii^	0.99	2.44	3.221 (2)	136
C202—H20*E*⋯Cl1^iii^	0.98	2.81	3.786 (3)	177
C221—H22*A*⋯Cl1^iv^	0.98	2.77	3.652 (2)	151

Symmetry codes: (i) 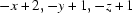; (ii) 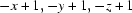; (iii) 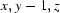; (iv) 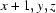.

**Fig. 1.**
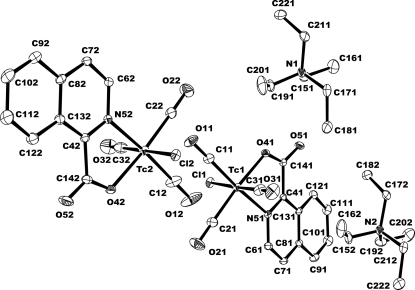
A view of the molecular structure of (I) with displacement ellipsoids for the non-hydrogen atoms drawn at the 30% probability level (H atoms omitted for clarity).

**Fig. 2.**
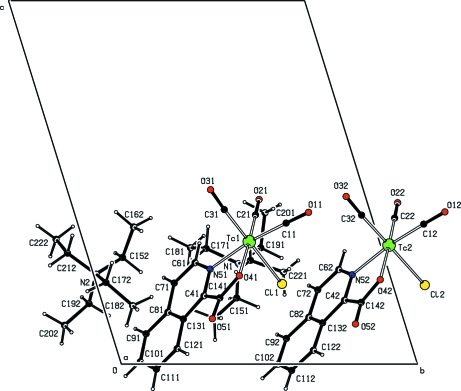
A view of the independent molecules down [100] indicating the superstructure effect (translation t) along the b-axis.

**Fig. 3.**
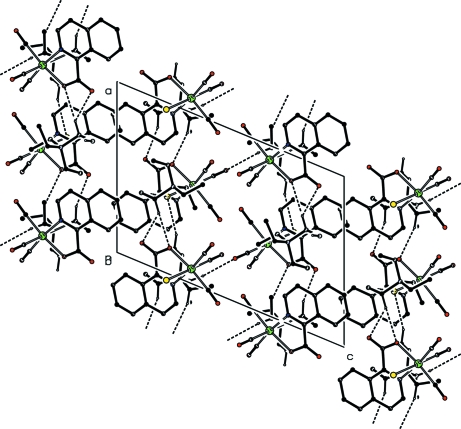
A view down [010] of a section of (I) with intermolecular hydrogen bonding contacts of the columns indicated by dashed lines.

## supplementary crystallographic information

### Comment

The chemistry of technetium is well established for diagnostic purposes, where tricarbonyl Tc-99*m* and Tc-99*m* dicarbonyl-nitrosyl complexes are employed (Alberto *et al.*, 1996; Rattat *et al.*, 2001). The title compound, (I), is one of several synthesized using isochinolinic acid under a variety of ligands in order to study their coordination or complexation properties (Alberto *et al.* 1996). Clinical or biological test results are summarized in Waibel *et al.* (1999).

A careful check for missed symmetry in structures that crystallize in space group P1 with *Z*' = 2 is always good practice, as can be judged in two out of many critical articles: in his classic review, Marsh (1995) detected about 20 structures (described in space group P1 with *Z* = 4) which were shown to be better described by the monoclinic space groups No. 14 and 15; and some 60 space-group corrections have been reported (Marsh *et al.* 2002) of which four displayed the same missed symmetry. In (I), a pseudo-inversion centre (Fig. 1) and a pseudo-translation (Fig. 2) were found during the checking process using the programme *PLATON* (Spek 2003).

The independent Tc complexes are related by a non-crystallographic inversion centre located at (3/4, 3/4, 1/2) which is equivalent to a pseudotranslation t at (1/2, 1/2, 1) and is interpreted as a superstructure effect. The distribution of strong (h+k = 2n) and weak intensity reflections (h+k = 2n+1) in the (*hk*0) plane supports the view of a superlattice phenomenon (Fig. 2). Many homomolecular crystal structures were analysed with respect to non-crystallographic symmetry and superstructure effects, where only about 20 structures out of 1166 with *Z* = 4 showed local pseudo-centres of symmetry (Desiraju *et al.*, 1991). The title compound belongs to a similar, heteromolecular class of structures with pseudoinversion centres, *Z* = 4 and with space group P1.

The pseudo-inversion related carbonyl atoms are found to point towards each other. The isochinolato ligands in both Tc complexes have bidentate coordination to the Tc atom *via* the aromatic amine N and carboxylate O atoms. This ligand, together with the two carbonyl ligands forms a distorted square planar environment around the Tc atom. The Cl atom and another carbonyl ligand are positioned *trans* to one another and complete the octahedral coordination geometry. The Tc coordination distances and angles are in comparable ranges, e. g. for Tc—CO (1.914 (7) - 1.927 (6) Å, Alberto *et al.* 1995) and 1.894 (3) - 1.912 (3) Å Alberto *et al.* 1996). Corresponding distances in the title complex vary between 1.905 (2) and 1.916 (2) Å, with an average value of 1.910 Å (Table 1).

The hydrogen bonding contacts in (I) can be described with the graph set descriptors D, C and R (Etter *et al.* 1990; Bernstein *et al.* 1995), with tetraethylammonium cations N1 and N2 as donor and the Tc complexes Tc1 and Tc2 as acceptor units. The tetraethylammonium donor group N1 is linked to acceptor atoms O31 (carbonyl), Cl1 and both carboxyl atoms O41, O51 of complex Tc1 to form weak intermolecular C—H···O and C—H···Cl hydrogen bonds (Desiraju & Steiner, 1999) with the graph set pattern D (Bernstein *et al.* 1995). Similar C—H···O and C—H···Cl hydrogen bond parameters (Table 2) were reported for tetrakis(pyridine) platinum(II) chloride trihydrate (Steiner & Saenger, 1993). The donor group of tetraethylammonium N2 is linked to both Tc complexes: Atom Cl1 is a bifurcated acceptor in complex Tc1, and the carboxylate group in complex Tc2 is an acceptor for the three-centre donor H19C in cation N2 (Table 2, Figure 3).

The hydrogen bonding contacts C151—H15A···O51 and C191—H19B···O41 represent a ring motif *R*^2^_2_(8), whereas C171—H17A···O31 and C221—H22A···Cl1 are linked to form a chain motif C^2^_2_(10). The contact C192—H19C···O52 can be seen as a simple motif D, or, together with the much weaker contact C192—H19C···O42 as a ring motif *R*^2^_1_(4). The link C202—H20E···Cl1 may be considered as a very weak hydrogen bonding contact with motif D. The combination of the hydrogen bonding motifs form ladder-like columns with carbonyl and chloro units in the centre and isochinolato planes outside, the latter being able to form partial π···π stacking interactions between the independent rings C81 - C131 (*Cg*3) and N52, C42, C132, C82, C72, C62 (*Cg*6), with distances between centroids *Cg*3 and *Cg*6 = 4.3733 (11) Å, dihedral angle between ring planes = 6.83 °, and a slippage of 3.214 Å.

### Experimental

Caution! Tc-99 is a weak *β*-emitter with a half life of 2.12 × 10^5^ years. Although radiation from low amounts of material is absorbed completely by the glass walls, reactions should only be carried out in specially equipped laboratories and under well ventilated hoods to avoid contamination or ingestion. Synthesis of the adduct [NEt_4_]_2_[TcCl_3_(CO)_3_] was prepared as described previously (Alberto *et al.* 1996). [NEt_4_]_2_[TcCl_3_(CO)_3_] was dissolved in methanol and 1 equivalent of isoquinolinic acid added to the solution. Stirring at room temperatur for about 4 h resulted in the quantitative formation of the title compound as observed from HPLC monitoring. The colour of the solvent turned to yellow. Methanol was evaporated and the residue taken up in THF. Slow evaporation of the THF gave yellow plates of (I) of good *x*-ray quality.

### Refinement

The missing cusp of data (alert level A in the *PLATON* checkcif) is due to data collection by rotation around the spindle axis only. This was standard at the time when the data were collected (in 1998) on a Stoe *IPDS1* image-plate system.

All the hydrogen atoms were geometrically placed (C—H = 0.95-0.99 Å) and refined as riding with *U*_iso_(H) = 1.2(*U*_eq_(C) or 1.5U_eq_(methyl C).

### Crystal data

**Table:**

(C_8_H_20_N)[Tc(C_10_H_6_NO_2_)Cl(CO)_3_]	*Z* = 4
*M**_r_* = 520.80	*F*_000_ = 1064
Triclinic, *P*1	*D*_x_ = 1.511 Mg m^−^^3^
Hall symbol: -P 1	Mo *K*α radiation λ = 0.71073 Å
*a* = 11.7657 (14) Å	Cell parameters from 5000 reflections
*b* = 12.7481 (14) Å	θ = 2.3–30.5º
*c* = 17.1855 (18) Å	µ = 0.78 mm^−^^1^
α = 102.878 (12)º	*T* = 193 (2) K
β = 109.624 (12)º	Irregular plate, yellow
γ = 99.052 (13)º	0.77 × 0.48 × 0.19 mm
*V* = 2290.0 (5) Å^3^	

### Data collection

**Table:**

Stoe IPDS diffractometer	12462 independent reflections
Radiation source: fine-focus sealed tube	9515 reflections with *I* > 2σ(*I*)
Monochromator: graphite	*R*_int_ = 0.058
*T* = 193(2) K	θ_max_ = 30.3º
φ rotation scan	θ_min_ = 2.9º
Absorption correction: numerical(Coppens *et al.*, 1965)	*h* = −16→15
*T*_min_ = 0.670, *T*_max_ = 0.828	*k* = −17→17
29670 measured reflections	*l* = −24→24

### Refinement

**Table:**

Refinement on *F*^2^	Secondary atom site location: difference Fourier map
Least-squares matrix: full	Hydrogen site location: inferred from neighbouring sites
*R*[*F*^2^ > 2σ(*F*^2^)] = 0.028	H-atom parameters constrained
*wR*(*F*^2^) = 0.067	*w* = 1/[σ^2^(*F*_o_^2^) + (0.04*P*)^2^] where *P* = (*F*_o_^2^ + 2*F*_c_^2^)/3
*S* = 1.00	(Δ/σ)_max_ = 0.003
12462 reflections	Δρ_max_ = 0.54 e Å^−^^3^
541 parameters	Δρ_min_ = −1.17 e Å^−^^3^
Primary atom site location: structure-invariant direct methods	Extinction correction: none

### Special details

**Table:**

Experimental. Due to the large crystal size a collimator of 0.80 mm diameter was used for the X-ray experiment.
Geometry. All e.s.d.'s (except the e.s.d. in the dihedral angle between two l.s. planes) are estimated using the full covariance matrix. The cell e.s.d.'s are taken into account individually in the estimation of e.s.d.'s in distances, angles and torsion angles; correlations between e.s.d.'s in cell parameters are only used when they are defined by crystal symmetry. An approximate (isotropic) treatment of cell e.s.d.'s is used for estimating e.s.d.'s involving l.s. planes.
Refinement. Refinement of *F*^2^ against ALL reflections. The weighted *R*-factor *wR* and goodness of fit *S* are based on *F*^2^, conventional *R*-factors *R* are based on *F*, with *F* set to zero for negative *F*^2^. The threshold expression of *F*^2^ > σ(*F*^2^) is used only for calculating *R*-factors(gt) *etc*. and is not relevant to the choice of reflections for refinement. *R*-factors based on *F*^2^ are statistically about twice as large as those based on *F*, and *R*- factors based on ALL data will be even larger.

### Fractional atomic coordinates and isotropic or equivalent isotropic displacement parameters (Å^2^)

**Table:**

	*x*	*y*	*z*	*U*_iso_*/*U*_eq_	
Tc1	0.579938 (12)	0.560390 (12)	0.336924 (8)	0.02087 (4)	
Tc2	0.915007 (12)	0.971138 (13)	0.672618 (8)	0.02372 (4)	
Cl1	0.44939 (4)	0.62682 (4)	0.22082 (3)	0.02909 (9)	
Cl2	1.04216 (4)	0.89237 (4)	0.78263 (3)	0.02847 (9)	
C11	0.69463 (17)	0.70442 (17)	0.38802 (11)	0.0301 (4)	
O11	0.76137 (16)	0.79139 (14)	0.41766 (9)	0.0480 (4)	
C21	0.49644 (17)	0.60941 (18)	0.41044 (12)	0.0333 (4)	
O21	0.44378 (16)	0.63781 (18)	0.45294 (11)	0.0629 (6)	
C31	0.68419 (18)	0.50959 (19)	0.42515 (12)	0.0344 (4)	
O31	0.74654 (17)	0.48412 (17)	0.47975 (11)	0.0562 (5)	
O41	0.65007 (10)	0.49125 (11)	0.24332 (8)	0.0247 (3)	
O51	0.61395 (12)	0.35498 (12)	0.12502 (9)	0.0332 (3)	
C41	0.46026 (13)	0.35068 (14)	0.18788 (10)	0.0182 (3)	
N51	0.44924 (12)	0.39932 (12)	0.26145 (8)	0.0193 (3)	
C61	0.35027 (15)	0.35543 (15)	0.27899 (11)	0.0233 (3)	
H61	0.3448	0.3903	0.3321	0.028*	
C71	0.25919 (15)	0.26352 (15)	0.22308 (11)	0.0238 (3)	
H71	0.1924	0.2344	0.2378	0.029*	
C81	0.26467 (14)	0.21165 (15)	0.14299 (10)	0.0215 (3)	
C91	0.17206 (16)	0.11541 (17)	0.08350 (12)	0.0299 (4)	
H91	0.1046	0.0848	0.0970	0.036*	
C101	0.17987 (17)	0.06671 (18)	0.00664 (12)	0.0344 (4)	
H101	0.1185	0.0016	−0.0328	0.041*	
C111	0.27871 (17)	0.11307 (17)	−0.01395 (12)	0.0313 (4)	
H111	0.2810	0.0801	−0.0686	0.038*	
C121	0.37207 (16)	0.20468 (16)	0.04271 (10)	0.0252 (4)	
H121	0.4389	0.2333	0.0278	0.030*	
C131	0.36789 (14)	0.25651 (14)	0.12404 (10)	0.0189 (3)	
C141	0.58280 (14)	0.40184 (15)	0.18218 (11)	0.0216 (3)	
C12	0.7925 (2)	0.8320 (2)	0.61471 (12)	0.0460 (6)	
O12	0.7211 (2)	0.74774 (19)	0.58124 (10)	0.0817 (8)	
C22	0.99797 (19)	0.92389 (18)	0.59816 (12)	0.0347 (5)	
O22	1.05157 (18)	0.89761 (17)	0.55594 (12)	0.0606 (5)	
C32	0.81724 (19)	1.0325 (2)	0.58993 (13)	0.0408 (5)	
O32	0.75712 (18)	1.0651 (2)	0.53876 (13)	0.0663 (6)	
O42	0.84573 (11)	1.03714 (11)	0.76777 (8)	0.0273 (3)	
O52	0.88396 (15)	1.16706 (15)	0.88951 (11)	0.0542 (5)	
C42	1.03499 (14)	1.17919 (15)	0.82419 (10)	0.0213 (3)	
N52	1.05141 (12)	1.12656 (12)	0.75407 (9)	0.0207 (3)	
C62	1.15557 (15)	1.16634 (16)	0.73994 (11)	0.0247 (3)	
H62	1.1654	1.1279	0.6894	0.030*	
C72	1.24548 (15)	1.25891 (16)	0.79563 (12)	0.0252 (4)	
H72	1.3168	1.2841	0.7841	0.030*	
C82	1.23181 (15)	1.31745 (15)	0.87097 (11)	0.0233 (3)	
C92	1.32207 (17)	1.41574 (17)	0.92933 (12)	0.0312 (4)	
H92	1.3939	1.4425	0.9188	0.037*	
C102	1.3058 (2)	1.47170 (19)	1.00045 (13)	0.0392 (5)	
H102	1.3657	1.5382	1.0390	0.047*	
C112	1.2008 (2)	1.4316 (2)	1.01720 (13)	0.0411 (5)	
H112	1.1919	1.4708	1.0678	0.049*	
C122	1.11075 (18)	1.33701 (18)	0.96199 (12)	0.0332 (4)	
H122	1.0401	1.3118	0.9743	0.040*	
C132	1.12380 (15)	1.27707 (16)	0.88639 (11)	0.0236 (3)	
C142	0.91312 (15)	1.12532 (17)	0.82997 (12)	0.0273 (4)	
N1	1.00717 (13)	0.50091 (13)	0.27668 (10)	0.0278 (3)	
C151	0.91776 (17)	0.45915 (18)	0.18202 (13)	0.0330 (4)	
H15A	0.8319	0.4364	0.1795	0.040*	
H15B	0.9214	0.5218	0.1569	0.040*	
C161	0.9424 (3)	0.3628 (2)	0.12612 (15)	0.0511 (6)	
H16A	0.8807	0.3421	0.0667	0.077*	
H16B	0.9363	0.2991	0.1490	0.077*	
H16C	1.0263	0.3847	0.1265	0.077*	
C171	1.00937 (18)	0.40781 (17)	0.31826 (13)	0.0327 (4)	
H17A	1.0720	0.4372	0.3784	0.039*	
H17B	1.0372	0.3490	0.2866	0.039*	
C181	0.8857 (2)	0.3555 (2)	0.32025 (16)	0.0450 (5)	
H18A	0.8958	0.2966	0.3483	0.067*	
H18B	0.8233	0.3236	0.2610	0.067*	
H18C	0.8580	0.4124	0.3528	0.067*	
C191	0.96073 (18)	0.59276 (18)	0.32010 (14)	0.0364 (5)	
H19A	0.9631	0.6524	0.2919	0.044*	
H19B	0.8725	0.5622	0.3100	0.044*	
C201	1.0336 (3)	0.6438 (2)	0.41647 (18)	0.0641 (9)	
H20A	0.9978	0.7023	0.4388	0.096*	
H20B	1.1208	0.6759	0.4274	0.096*	
H20C	1.0293	0.5862	0.4456	0.096*	
C211	1.13983 (17)	0.54488 (19)	0.28542 (17)	0.0437 (6)	
H21A	1.1955	0.5655	0.3472	0.052*	
H21B	1.1649	0.4840	0.2535	0.052*	
C221	1.1606 (2)	0.6439 (2)	0.2530 (2)	0.0666 (9)	
H22A	1.2485	0.6663	0.2613	0.100*	
H22B	1.1386	0.7057	0.2852	0.100*	
H22C	1.1082	0.6241	0.1913	0.100*	
N2	0.52263 (13)	0.00906 (14)	0.24769 (10)	0.0265 (3)	
C152	0.5005 (2)	0.11587 (19)	0.29338 (14)	0.0386 (5)	
H15C	0.4839	0.1620	0.2535	0.046*	
H15D	0.4246	0.0966	0.3055	0.046*	
C162	0.6065 (3)	0.1853 (2)	0.37740 (17)	0.0603 (8)	
H16D	0.5838	0.2521	0.4019	0.091*	
H16E	0.6818	0.2069	0.3663	0.091*	
H16F	0.6222	0.1416	0.4184	0.091*	
C172	0.63615 (16)	0.03339 (18)	0.22472 (14)	0.0323 (4)	
H17C	0.7094	0.0745	0.2786	0.039*	
H17D	0.6522	−0.0382	0.2005	0.039*	
C182	0.6258 (2)	0.0996 (2)	0.16093 (17)	0.0437 (5)	
H18D	0.7028	0.1110	0.1501	0.065*	
H18E	0.6126	0.1719	0.1848	0.065*	
H18F	0.5551	0.0589	0.1065	0.065*	
C192	0.40524 (17)	−0.0458 (2)	0.16643 (13)	0.0374 (5)	
H19C	0.3341	−0.0630	0.1840	0.045*	
H19D	0.3891	0.0084	0.1334	0.045*	
C202	0.4084 (3)	−0.1512 (2)	0.10697 (16)	0.0547 (7)	
H20D	0.3291	−0.1800	0.0568	0.082*	
H20E	0.4214	−0.2068	0.1381	0.082*	
H20F	0.4768	−0.1352	0.0875	0.082*	
C212	0.54784 (19)	−0.06576 (18)	0.30546 (14)	0.0347 (4)	
H21C	0.5531	−0.1373	0.2715	0.042*	
H21D	0.6301	−0.0308	0.3534	0.042*	
C222	0.4516 (3)	−0.0895 (3)	0.34348 (19)	0.0544 (6)	
H22D	0.4754	−0.1379	0.3800	0.082*	
H22E	0.3701	−0.1267	0.2967	0.082*	
H22F	0.4469	−0.0195	0.3785	0.082*	

### Atomic displacement parameters (Å^2^)

**Table:**

	*U*^11^	*U*^22^	*U*^33^	*U*^12^	*U*^13^	*U*^23^
Tc1	0.01960 (6)	0.02175 (8)	0.01644 (6)	−0.00022 (5)	0.00534 (5)	0.00280 (5)
Tc2	0.02287 (7)	0.02603 (9)	0.01804 (6)	−0.00056 (5)	0.00705 (5)	0.00450 (5)
Cl1	0.0306 (2)	0.0260 (2)	0.02687 (19)	0.00637 (16)	0.00671 (16)	0.00788 (16)
Cl2	0.0301 (2)	0.0237 (2)	0.0297 (2)	0.00575 (15)	0.01008 (16)	0.00745 (17)
C11	0.0328 (9)	0.0322 (11)	0.0183 (7)	−0.0019 (7)	0.0091 (7)	0.0028 (7)
O11	0.0589 (10)	0.0381 (10)	0.0285 (7)	−0.0197 (7)	0.0154 (7)	−0.0010 (6)
C21	0.0300 (9)	0.0352 (12)	0.0264 (8)	−0.0024 (7)	0.0121 (7)	−0.0011 (8)
O21	0.0514 (10)	0.0769 (14)	0.0480 (9)	−0.0003 (9)	0.0319 (8)	−0.0136 (9)
C31	0.0308 (9)	0.0371 (12)	0.0239 (8)	−0.0036 (7)	0.0034 (7)	0.0069 (8)
O31	0.0511 (10)	0.0611 (13)	0.0442 (9)	0.0115 (8)	−0.0011 (8)	0.0246 (9)
O41	0.0192 (5)	0.0235 (7)	0.0287 (6)	0.0003 (4)	0.0115 (5)	0.0028 (5)
O51	0.0310 (6)	0.0288 (8)	0.0399 (7)	0.0010 (5)	0.0240 (6)	−0.0009 (6)
C41	0.0177 (6)	0.0179 (8)	0.0209 (7)	0.0047 (5)	0.0081 (5)	0.0085 (6)
N51	0.0181 (6)	0.0185 (7)	0.0212 (6)	0.0032 (5)	0.0079 (5)	0.0058 (5)
C61	0.0243 (7)	0.0243 (9)	0.0240 (7)	0.0042 (6)	0.0132 (6)	0.0078 (7)
C71	0.0193 (7)	0.0258 (10)	0.0288 (8)	0.0029 (6)	0.0121 (6)	0.0106 (7)
C81	0.0167 (7)	0.0217 (9)	0.0245 (7)	0.0026 (6)	0.0060 (6)	0.0087 (6)
C91	0.0216 (8)	0.0291 (11)	0.0314 (9)	−0.0026 (6)	0.0060 (7)	0.0073 (7)
C101	0.0275 (9)	0.0313 (11)	0.0277 (8)	−0.0028 (7)	0.0004 (7)	−0.0001 (8)
C111	0.0341 (9)	0.0293 (11)	0.0232 (8)	0.0049 (7)	0.0070 (7)	0.0022 (7)
C121	0.0264 (8)	0.0263 (10)	0.0213 (7)	0.0050 (6)	0.0092 (6)	0.0051 (7)
C131	0.0182 (7)	0.0187 (9)	0.0206 (7)	0.0052 (5)	0.0069 (5)	0.0081 (6)
C141	0.0202 (7)	0.0203 (9)	0.0275 (7)	0.0053 (6)	0.0118 (6)	0.0092 (6)
C12	0.0496 (12)	0.0523 (15)	0.0166 (8)	−0.0203 (10)	0.0094 (8)	0.0014 (8)
O12	0.0981 (16)	0.0747 (15)	0.0267 (8)	−0.0562 (12)	0.0153 (9)	−0.0035 (8)
C22	0.0405 (10)	0.0305 (11)	0.0290 (9)	0.0006 (8)	0.0171 (8)	0.0008 (8)
O22	0.0698 (12)	0.0578 (12)	0.0545 (10)	0.0054 (9)	0.0429 (9)	−0.0048 (9)
C32	0.0316 (10)	0.0588 (16)	0.0328 (10)	0.0070 (9)	0.0120 (8)	0.0188 (10)
O32	0.0552 (11)	0.1014 (18)	0.0556 (11)	0.0295 (11)	0.0165 (9)	0.0495 (12)
O42	0.0217 (6)	0.0298 (7)	0.0297 (6)	0.0014 (5)	0.0132 (5)	0.0057 (5)
O52	0.0441 (8)	0.0552 (11)	0.0542 (9)	−0.0112 (7)	0.0374 (8)	−0.0134 (8)
C42	0.0205 (7)	0.0222 (9)	0.0242 (7)	0.0056 (6)	0.0104 (6)	0.0095 (6)
N52	0.0211 (6)	0.0192 (8)	0.0251 (6)	0.0055 (5)	0.0119 (5)	0.0080 (5)
C62	0.0268 (8)	0.0222 (9)	0.0313 (8)	0.0072 (6)	0.0175 (7)	0.0094 (7)
C72	0.0230 (7)	0.0247 (10)	0.0348 (9)	0.0068 (6)	0.0157 (7)	0.0143 (7)
C82	0.0217 (7)	0.0229 (9)	0.0253 (7)	0.0039 (6)	0.0065 (6)	0.0122 (7)
C92	0.0276 (8)	0.0305 (11)	0.0295 (9)	−0.0008 (7)	0.0053 (7)	0.0122 (8)
C102	0.0398 (11)	0.0349 (12)	0.0268 (9)	−0.0066 (8)	0.0038 (8)	0.0037 (8)
C112	0.0482 (12)	0.0400 (13)	0.0254 (9)	−0.0003 (9)	0.0139 (8)	−0.0005 (8)
C122	0.0348 (9)	0.0362 (12)	0.0248 (8)	0.0016 (8)	0.0139 (7)	0.0034 (8)
C132	0.0233 (7)	0.0257 (10)	0.0227 (7)	0.0054 (6)	0.0090 (6)	0.0090 (7)
C142	0.0229 (8)	0.0301 (10)	0.0314 (8)	0.0040 (6)	0.0156 (7)	0.0081 (7)
N1	0.0200 (6)	0.0227 (8)	0.0375 (8)	0.0081 (5)	0.0071 (6)	0.0072 (6)
C151	0.0270 (9)	0.0347 (12)	0.0353 (9)	0.0060 (7)	0.0084 (7)	0.0136 (8)
C161	0.0666 (16)	0.0513 (16)	0.0354 (11)	0.0166 (12)	0.0216 (11)	0.0086 (10)
C171	0.0367 (10)	0.0262 (11)	0.0335 (9)	0.0137 (8)	0.0089 (8)	0.0088 (8)
C181	0.0565 (14)	0.0368 (13)	0.0534 (13)	0.0159 (10)	0.0301 (11)	0.0192 (11)
C191	0.0290 (9)	0.0262 (11)	0.0492 (11)	0.0130 (7)	0.0104 (8)	0.0050 (9)
C201	0.0681 (17)	0.0443 (16)	0.0562 (15)	0.0240 (13)	0.0073 (13)	−0.0114 (13)
C211	0.0189 (8)	0.0355 (12)	0.0734 (15)	0.0099 (7)	0.0139 (9)	0.0137 (11)
C221	0.0342 (12)	0.0507 (17)	0.130 (3)	0.0111 (11)	0.0415 (15)	0.0396 (18)
N2	0.0226 (7)	0.0250 (8)	0.0322 (7)	0.0090 (5)	0.0091 (6)	0.0093 (6)
C152	0.0523 (12)	0.0318 (12)	0.0452 (11)	0.0260 (9)	0.0247 (10)	0.0173 (9)
C162	0.092 (2)	0.0358 (15)	0.0455 (13)	0.0228 (13)	0.0204 (13)	0.0021 (11)
C172	0.0224 (8)	0.0306 (11)	0.0472 (11)	0.0112 (7)	0.0141 (8)	0.0138 (9)
C182	0.0364 (11)	0.0413 (14)	0.0671 (15)	0.0133 (9)	0.0270 (10)	0.0289 (11)
C192	0.0217 (8)	0.0470 (14)	0.0375 (10)	0.0022 (8)	0.0065 (7)	0.0133 (9)
C202	0.0623 (16)	0.0413 (15)	0.0414 (12)	−0.0055 (11)	0.0102 (11)	0.0039 (11)
C212	0.0404 (10)	0.0274 (11)	0.0404 (10)	0.0141 (8)	0.0147 (8)	0.0153 (8)
C222	0.0690 (17)	0.0512 (17)	0.0658 (16)	0.0214 (13)	0.0411 (14)	0.0325 (13)

### Geometric parameters (Å, °)

**Table:**

Tc1—C21	1.9045 (18)	C122—H122	0.9500
Tc1—C31	1.913 (2)	N1—C171	1.515 (3)
Tc1—C11	1.916 (2)	N1—C211	1.517 (2)
Tc1—O41	2.1293 (12)	N1—C191	1.521 (2)
Tc1—N51	2.1778 (15)	N1—C151	1.525 (2)
Tc1—Cl1	2.4822 (6)	C151—C161	1.513 (3)
Tc2—C22	1.9060 (19)	C151—H15A	0.9900
Tc2—C32	1.907 (2)	C151—H15B	0.9900
Tc2—C12	1.913 (2)	C161—H16A	0.9800
Tc2—O42	2.1317 (12)	C161—H16B	0.9800
Tc2—N52	2.1714 (15)	C161—H16C	0.9800
Tc2—Cl2	2.4980 (6)	C171—C181	1.517 (3)
C11—O11	1.145 (2)	C171—H17A	0.9900
C21—O21	1.142 (2)	C171—H17B	0.9900
C31—O31	1.128 (3)	C181—H18A	0.9800
O41—C141	1.286 (2)	C181—H18B	0.9800
O51—C141	1.227 (2)	C181—H18C	0.9800
C41—N51	1.3363 (19)	C191—C201	1.514 (3)
C41—C131	1.424 (2)	C191—H19A	0.9900
C41—C141	1.530 (2)	C191—H19B	0.9900
N51—C61	1.366 (2)	C201—H20A	0.9800
C61—C71	1.362 (2)	C201—H20B	0.9800
C61—H61	0.9500	C201—H20C	0.9800
C71—C81	1.415 (2)	C211—C221	1.508 (4)
C71—H71	0.9500	C211—H21A	0.9900
C81—C91	1.418 (2)	C211—H21B	0.9900
C81—C131	1.428 (2)	C221—H22A	0.9800
C91—C101	1.368 (3)	C221—H22B	0.9800
C91—H91	0.9500	C221—H22C	0.9800
C101—C111	1.407 (3)	N2—C212	1.516 (3)
C101—H101	0.9500	N2—C152	1.520 (3)
C111—C121	1.375 (2)	N2—C192	1.521 (2)
C111—H111	0.9500	N2—C172	1.521 (2)
C121—C131	1.427 (2)	C152—C162	1.515 (3)
C121—H121	0.9500	C152—H15C	0.9900
C12—O12	1.142 (3)	C152—H15D	0.9900
C22—O22	1.141 (2)	C162—H16D	0.9800
C32—O32	1.133 (3)	C162—H16E	0.9800
O42—C142	1.284 (2)	C162—H16F	0.9800
O52—C142	1.224 (2)	C172—C182	1.512 (3)
C42—N52	1.333 (2)	C172—H17C	0.9900
C42—C132	1.427 (2)	C172—H17D	0.9900
C42—C142	1.534 (2)	C182—H18D	0.9800
N52—C62	1.370 (2)	C182—H18E	0.9800
C62—C72	1.361 (2)	C182—H18F	0.9800
C62—H62	0.9500	C192—C202	1.510 (3)
C72—C82	1.416 (2)	C192—H19C	0.9900
C72—H72	0.9500	C192—H19D	0.9900
C82—C92	1.418 (2)	C202—H20D	0.9800
C82—C132	1.425 (2)	C202—H20E	0.9800
C92—C102	1.360 (3)	C202—H20F	0.9800
C92—H92	0.9500	C212—C222	1.509 (3)
C102—C112	1.405 (3)	C212—H21C	0.9900
C102—H102	0.9500	C212—H21D	0.9900
C112—C122	1.375 (3)	C222—H22D	0.9800
C112—H112	0.9500	C222—H22E	0.9800
C122—C132	1.423 (2)	C222—H22F	0.9800
			
C21—Tc1—C31	89.43 (9)	C211—N1—C191	110.81 (16)
C21—Tc1—C11	87.48 (8)	C171—N1—C151	111.09 (15)
C31—Tc1—C11	89.41 (9)	C211—N1—C151	111.14 (17)
C21—Tc1—O41	172.44 (6)	C191—N1—C151	105.93 (14)
C31—Tc1—O41	94.13 (7)	C161—C151—N1	115.46 (17)
C11—Tc1—O41	99.20 (6)	C161—C151—H15A	108.4
C21—Tc1—N51	98.23 (7)	N1—C151—H15A	108.4
C31—Tc1—N51	96.33 (7)	C161—C151—H15B	108.4
C11—Tc1—N51	171.91 (6)	N1—C151—H15B	108.4
O41—Tc1—N51	74.77 (5)	H15A—C151—H15B	107.5
C21—Tc1—Cl1	92.04 (7)	C151—C161—H16A	109.5
C31—Tc1—Cl1	178.38 (6)	C151—C161—H16B	109.5
C11—Tc1—Cl1	89.97 (6)	H16A—C161—H16B	109.5
O41—Tc1—Cl1	84.49 (4)	C151—C161—H16C	109.5
N51—Tc1—Cl1	84.14 (4)	H16A—C161—H16C	109.5
C22—Tc2—C32	90.02 (10)	H16B—C161—H16C	109.5
C22—Tc2—C12	87.83 (9)	N1—C171—C181	115.07 (17)
C32—Tc2—C12	88.76 (11)	N1—C171—H17A	108.5
C22—Tc2—O42	172.46 (7)	C181—C171—H17A	108.5
C32—Tc2—O42	93.54 (8)	N1—C171—H17B	108.5
C12—Tc2—O42	98.88 (8)	C181—C171—H17B	108.5
C22—Tc2—N52	98.15 (7)	H17A—C171—H17B	107.5
C32—Tc2—N52	96.01 (9)	C171—C181—H18A	109.5
C12—Tc2—N52	172.34 (7)	C171—C181—H18B	109.5
O42—Tc2—N52	74.89 (5)	H18A—C181—H18B	109.5
C22—Tc2—Cl2	90.81 (7)	C171—C181—H18C	109.5
C32—Tc2—Cl2	179.14 (7)	H18A—C181—H18C	109.5
C12—Tc2—Cl2	91.51 (8)	H18B—C181—H18C	109.5
O42—Tc2—Cl2	85.61 (4)	C201—C191—N1	115.09 (17)
N52—Tc2—Cl2	83.64 (4)	C201—C191—H19A	108.5
O11—C11—Tc1	178.6 (2)	N1—C191—H19A	108.5
O21—C21—Tc1	178.41 (18)	C201—C191—H19B	108.5
O31—C31—Tc1	176.9 (2)	N1—C191—H19B	108.5
C141—O41—Tc1	119.02 (10)	H19A—C191—H19B	107.5
N51—C41—C131	121.89 (14)	C191—C201—H20A	109.5
N51—C41—C141	113.66 (13)	C191—C201—H20B	109.5
C131—C41—C141	124.37 (13)	H20A—C201—H20B	109.5
C41—N51—C61	119.94 (14)	C191—C201—H20C	109.5
C41—N51—Tc1	115.46 (11)	H20A—C201—H20C	109.5
C61—N51—Tc1	123.96 (11)	H20B—C201—H20C	109.5
C71—C61—N51	122.38 (15)	C221—C211—N1	115.40 (17)
C71—C61—H61	118.8	C221—C211—H21A	108.4
N51—C61—H61	118.8	N1—C211—H21A	108.4
C61—C71—C81	119.62 (15)	C221—C211—H21B	108.4
C61—C71—H71	120.2	N1—C211—H21B	108.4
C81—C71—H71	120.2	H21A—C211—H21B	107.5
C71—C81—C91	121.18 (15)	C211—C221—H22A	109.5
C71—C81—C131	118.52 (14)	C211—C221—H22B	109.5
C91—C81—C131	120.28 (15)	H22A—C221—H22B	109.5
C101—C91—C81	119.97 (17)	C211—C221—H22C	109.5
C101—C91—H91	120.0	H22A—C221—H22C	109.5
C81—C91—H91	120.0	H22B—C221—H22C	109.5
C91—C101—C111	120.04 (16)	C212—N2—C152	110.98 (15)
C91—C101—H101	120.0	C212—N2—C192	111.54 (16)
C111—C101—H101	120.0	C152—N2—C192	105.92 (15)
C121—C111—C101	121.87 (17)	C212—N2—C172	106.74 (14)
C121—C111—H111	119.1	C152—N2—C172	111.04 (16)
C101—C111—H111	119.1	C192—N2—C172	110.70 (15)
C111—C121—C131	119.52 (16)	C162—C152—N2	115.38 (18)
C111—C121—H121	120.2	C162—C152—H15C	108.4
C131—C121—H121	120.2	N2—C152—H15C	108.4
C41—C131—C121	124.19 (14)	C162—C152—H15D	108.4
C41—C131—C81	117.57 (14)	N2—C152—H15D	108.4
C121—C131—C81	118.25 (14)	H15C—C152—H15D	107.5
O51—C141—O41	124.48 (15)	C152—C162—H16D	109.5
O51—C141—C41	120.41 (15)	C152—C162—H16E	109.5
O41—C141—C41	115.00 (13)	H16D—C162—H16E	109.5
O12—C12—Tc2	178.4 (3)	C152—C162—H16F	109.5
O22—C22—Tc2	177.57 (18)	H16D—C162—H16F	109.5
O32—C32—Tc2	177.4 (2)	H16E—C162—H16F	109.5
C142—O42—Tc2	119.15 (10)	C182—C172—N2	115.37 (15)
N52—C42—C132	121.81 (14)	C182—C172—H17C	108.4
N52—C42—C142	113.73 (14)	N2—C172—H17C	108.4
C132—C42—C142	124.46 (14)	C182—C172—H17D	108.4
C42—N52—C62	120.06 (14)	N2—C172—H17D	108.4
C42—N52—Tc2	116.62 (11)	H17C—C172—H17D	107.5
C62—N52—Tc2	123.11 (11)	C172—C182—H18D	109.5
C72—C62—N52	122.39 (15)	C172—C182—H18E	109.5
C72—C62—H62	118.8	H18D—C182—H18E	109.5
N52—C62—H62	118.8	C172—C182—H18F	109.5
C62—C72—C82	119.44 (15)	H18D—C182—H18F	109.5
C62—C72—H72	120.3	H18E—C182—H18F	109.5
C82—C72—H72	120.3	C202—C192—N2	115.64 (18)
C72—C82—C92	120.99 (16)	C202—C192—H19C	108.4
C72—C82—C132	118.75 (15)	N2—C192—H19C	108.4
C92—C82—C132	120.25 (16)	C202—C192—H19D	108.4
C102—C92—C82	119.95 (18)	N2—C192—H19D	108.4
C102—C92—H92	120.0	H19C—C192—H19D	107.4
C82—C92—H92	120.0	C192—C202—H20D	109.5
C92—C102—C112	120.34 (18)	C192—C202—H20E	109.5
C92—C102—H102	119.8	H20D—C202—H20E	109.5
C112—C102—H102	119.8	C192—C202—H20F	109.5
C122—C112—C102	121.54 (18)	H20D—C202—H20F	109.5
C122—C112—H112	119.2	H20E—C202—H20F	109.5
C102—C112—H112	119.2	C222—C212—N2	114.95 (18)
C112—C122—C132	119.69 (18)	C222—C212—H21C	108.5
C112—C122—H122	120.2	N2—C212—H21C	108.5
C132—C122—H122	120.2	C222—C212—H21D	108.5
C122—C132—C82	118.21 (16)	N2—C212—H21D	108.5
C122—C132—C42	124.23 (16)	H21C—C212—H21D	107.5
C82—C132—C42	117.55 (15)	C212—C222—H22D	109.5
O52—C142—O42	124.11 (16)	C212—C222—H22E	109.5
O52—C142—C42	120.75 (16)	H22D—C222—H22E	109.5
O42—C142—C42	115.12 (14)	C212—C222—H22F	109.5
C171—N1—C211	106.53 (15)	H22D—C222—H22F	109.5
C171—N1—C191	111.44 (16)	H22E—C222—H22F	109.5

### Hydrogen-bond geometry (Å, °)

**Table:**

*D*—H···*A*	*D*—H	H···*A*	*D*···*A*	*D*—H···*A*
C151—H15A···O51	0.99	2.38	3.328 (2)	161
C171—H17A···O31^i^	0.99	2.50	3.482 (3)	172
C191—H19B···O41	0.99	2.40	3.360 (2)	163
C192—H19C···O42^ii^	0.99	2.56	3.507 (2)	160
C192—H19C···O52^ii^	0.99	2.44	3.221 (2)	136
C202—H20E···Cl1^iii^	0.98	2.81	3.786 (3)	177
C221—H22A···Cl1^iv^	0.98	2.77	3.652 (2)	151

Symmetry codes: (i) −*x*+2, −*y*+1, −*z*+1; (ii) −*x*+1, −*y*+1, −*z*+1; (iii) *x*, *y*−1, *z*; (iv) *x*+1, *y*, *z*.
